# Applying a Precautionary Approach to Mobile Contact Tracing for COVID-19: The Value of Reversibility

**DOI:** 10.1007/s11673-020-10004-z

**Published:** 2020-08-25

**Authors:** Niels Nijsingh, Anne van Bergen, Verina Wild

**Affiliations:** Institute for Ethics, History and Theory of Medicine, Ludwig Maximilians Universität, Lessingstrasse 2, 80336 München, Germany

**Keywords:** Public health ethics, Risk, Precautionary principle, Pandemic, mHealth, Uncertainty, Infectious disease

## Abstract

The COVID-19 pandemic presents unprecedented challenges to public health decision-making. Specifically, the lack of evidence and the urgency with which a response is called for, raise the ethical challenge of assessing how much (and what kind of) evidence is required for the justification of interventions in response to the various threats we face. Here we discuss the intervention of introducing technology that aims to trace and alert contacts of infected persons—contact tracing (CT) technology. Determining whether such an intervention is proportional is complicated by complex trade-offs and feedback loops. We suggest that the resulting uncertainties necessitate a precautionary approach. On the one hand, precautionary reasons support CT technology as a means to contribute to the prevention of harms caused by alternative interventions, or COVID-19 itself. On the other hand, however, both the extent to which such technology itself present risks of serious harm, as well as its effectiveness, remain unclear. We therefore argue that a precautionary approach should put reversibility of CT technology at the forefront. We outline several practical implications.

It has been suggested that it could be morally justified, or even morally obligatory, to implement contact tracing technology (CT technology) in the response to the current COVID-19 pandemic (Parker et al. 2020). There are different ways of implementing CT technology, but they all aim to retrospectively trace and alert contacts of confirmed infected persons (Rimpiläinen, Thomas, and Morrison 2020). Like any other form of surveillance, CT technology raises privacy issues. However, given the enormous risks and burdens associated with either ongoing population lockdowns, or letting the virus spread freely, some infringement of privacy may be considered proportional (Schaefer and Ballantyne 2020). This raises the question of what an acceptable trade-off would be. What is a reasonable price to pay to help mitigate some of the worst effects of the global crisis?

## Epistemic Uncertainties

There are no easy answers to the question of the proportionality of CT technology or any of the other possible surveillance interventions. Attempts to deal with the various threats posed by the pandemic are hindered by epistemic uncertainties on different levels (Aven and Bouder 2020). The social, economic, and health risks associated with the pandemic are largely unknown and are systemically interrelated: in many cases addressing one risk will have effects on the probability and weight of the others (Hafiz et al. 2020). Worse still, there is not much room for experiments, the crisis is acute, and an overly hesitant approach may aggravate the situation, just as an overly rushed one could.

This challenge can be interpreted within a precautionary framework: lockdowns have been implemented across the globe by way of precaution against a rise in infection, which would lead to sickness, death, and potential overload of healthcare systems. However, since these lockdowns come at a great cost and present risks of their own (Hoffmann et al. 2020), questions arise on how long and to what extent these restrictions may be justified (Viens et al. 2009; Selgelid 2014). CT technology is presented as one tool to move beyond blanket lockdowns, contributing to the prevention of both the damage associated with COVID-19 directly and the consequences of responses to it (Parker 2020).

The complexity of the situation affects decision-making on proposed interventions. As we will illustrate, in determining the desirability of CT technology we are confronted by difficult trade-offs and feedback loops, the assessment of which is hindered by the overall epistemic uncertainty.

## Trade-offs and Feedback Loops

First, on the level of the *app itself*, there appears to be a trade-off between responsible data use and effectiveness. Responsible data use requires sufficient guarantees that data is stored and treated in a way that prevents abuse and protects privacy (Bock et al. 2020). For this reason, so-called decentralized approaches have been proposed as a way to protect users from the dangers of privacy infringement (Troncoso et al. 2020, see however Vaudenay 2020 for a discussion of the risks to privacy inherent to decentralized approaches). Increased privacy, however, is likely to decrease effectiveness. CT technology may be less potent as a public health tool when users and providers are unable to use identifying characteristics. For example, false positives may increase when the technology itself is unable to distinguish people who have been in close contact from those who were separated by a wall and there is no additional information available to put the results into context (European Commission 2020). These false positives risk further burdening an already overburdened healthcare system and might create anxieties or lead to unnecessary self-isolation. In addition, decentralized alternatives appear to be more vulnerable to trolling, where individuals purposefully feed the system with false information, as well as to plain human fallibility, where people accidentally do so. It appears that there may be good reasons from a public health perspective to store data in a way that provides access to aggregated and individualized data to track infection. Such mass storage of location data may be better suited to support analogous contact tracing practices, but it would also likely be prone to leaks and abuses. Furthermore, a centralized approach increases the risk of function creep, which occurs when data is used for other purposes than initially intended (Bock et al. 2020). Potentially, the infrastructure that is created in battling the pandemic enables governments, or private companies, to exercise an inordinate amount of control over individual citizens. For these reasons, a centralized approach needs a stronger justification in terms of a substantial increase of effectiveness, the evidence of which will be very difficult to establish.

Second, on the level of *societal implementation*, the complexities of CT technology can be illustrated by issues of uptake and voluntariness. Effectiveness can only be ensured if the uptake is sufficiently high (Cho, Ippolito and Yu 2020). However, not all citizens own a (sufficiently new) smartphone necessary to run an app (Gasser et al. 2020). Hardware may be distributed, in the form of keychains or cards, but this will be costly. Additionally, in a voluntary scheme, many people who do own a smartphone may refuse to install the app, because they (rightly or wrongly) fear that they may be hacked or controlled, because they do not wish to be forced to isolate, or because they think their contribution is not needed (Wong and Jensen 2020). If the perceived disadvantages are relatively high, motivation to participate will likely be low. This adds a layer to the trade-off between effectiveness and privacy discussed above. Especially if it’s suspected that there is a high number of false positives, and people would be isolating themselves “for nothing,” the individual costs might overshadow the perceived public benefits. The extent and weight of these disadvantages will depend on the wider context, which is formed by the availability of proper follow up, income protection insurance, and so on.

Trust in the prevailing system is another important factor (Ienca and Vayena 2020). However, trust may be negatively affected precisely by attempts to influence uptake, by either mandating participation or by incentivizing it. For example, the uptake may be increased if, as a counter-balance to the perceived disadvantages, there are relative advantages offered to the instalment and use of CT technology, for instance when the entrance to certain public spaces (restaurants, public transportation, stores) is conditional on participation (Lucivero 2020). These approaches need a stronger justification than voluntary ones, because they constitute a larger infringement of individual rights (EDPB 2020). Yet, they may be more difficult to justify for at least two reasons. Firstly, offering advantages will likely exacerbate inequalities. Even if we can arrange that everybody has access to the relevant technology, not everybody will be affected in the same way: people who are unable to work from home, who are in contact with many strangers, and who do not have the resources to easily self-isolate will be disproportionately affected. Secondly, (semi-) mandatory approaches may in some cases even create perverse incentives, for example when people are motivated to “chase immunity” in order to escape the burdens of the app. Any attempt to encourage or mandate CT apps is then likely to worsen the situation, by creating backlash effects that cause CT technology to lose support of large parts of the population (Munthe and Nijsingh 2019). It is precisely because of the potential benefit of being able to trace the spread of the virus more easily and pervasively than with the labour intensive method of contacting people personally that we may expect the chances of these kinds of effects to rise.

Attempts to responsibly introduce CT technology are thus confronted with feedback loops: low effectiveness raises costs and decreases uptake, attempts to counter this by raising effectiveness may decrease privacy, which then potentially decreases uptake, while raising uptake by implementing more or less mandatory approaches creates risks of backlash and crumbling public support, which then again lowers effectiveness. Of course, scenarios where positive reinforcing feedback loops take place are also possible. A fair and reliable system would lead to an increase in trust and potentially a shared feeling of solidarity, which will lead to a further increase in use and therefore effectiveness, etc. The problem is, we do not know the relative weight and probability of the different factors in play.

## Precautionary Principle

Due to the complexity and various types of uncertainties, any course of action, as well as abstaining from action, may potentially have grave consequences—while there is little time to thoroughly research the various options. This mix of uncertainty, complexity, and urgency raises the ethical challenge of assessing how much (and what kind of) evidence is required for the justification of interventions in response to the various threats we face. This is a familiar challenge in discussions on the precautionary principle (Steel 2015; Munthe 2011).

The precautionary principle (PP) is based on the tenet that uncertainty is not a sufficient reason to abstain from taking precautions to prevent some adverse outcome. Usually, responsible applications of PP will set lower standards of evidence to the extent that the potential damage that the precaution aims to prevent is serious and/or irreversible (Trouwborst 2009). This point of departure can be detailed in various ways, by balancing the expected adverse outcome to the precaution and the level of uncertainty (Steel 2015). One basic requirement to this balancing is *proportionality*. If PP is to be of any use as a practical guidance to responsible policy decision-making, it needs to be fleshed out in a way that is non-arbitrary and consistent. A non-arbitrary version of PP allows principled comparison between different precautions. It demands that when one precaution is preferred over another, this is done on the basis of generalizable reasons, for example those relating to costs, expected side effects or expected effectiveness. Consistency, moreover, demands that a version of PP that recommends a certain precaution does not at the same time recommend against that precaution. In a presently apt metaphor, the cure should not be worse than the disease (Singer and Plant 2020).

However, as we have seen, in complex messy contexts such as the COVID-19 pandemic, it is not always clear what proportionality demands (cf. Nijsingh forthcoming). The proportionality of CT technology will vary depending on contextual factors: How sensitive and specific are non-digital contact tracing measures and what risks are posed by these measures? What is the current level of community transmission of the virus? What is the general level of trust in infectious disease measures or in digital technology? Where is the CT technology introduced (dense cities or spread out rural areas)? Who decides whether CT technology is sufficiently effective and safe? Some of these contextual factors are dynamic, others are of a more permanent nature, some are local, some are universal and some are more or less empirically certain. Whether or not a CT technology will be justified depends on both which type exactly is being proposed and the context within which it is introduced. This makes it very difficult to predict whether CT technology will contribute to the solution, or rather make matters worse (cf. Morley et al. 2020). There is a strong imperative to conduct research in order to gather more evidence (Wild et al. 2020; Lucivero et al. 2020). But decisions need to be made now and even when more information is available, the situation will still be fraught with uncertainty. This uncertainty dramatically increases the chances of “getting it wrong.”

Our suggestion in this situation of epistemic uncertainty is not to abandon hope for a reasoned approach but to aim to keep one’s options open as much as possible. Since the current pandemic poses a multifaceted problem, there will be no “golden bullet” solution, rather some interventions may work well in some contexts or only in combination with specific other interventions. Note that, even though some of the trade-offs we described are specific to CT technology, uncertainty applies to many other interventions as well, including other types of contact tracing. These interventions pose risks of their own, which makes it even more difficult to give a balanced assessment of what the right course of action is likely to be. Given the lack of evidence, the need to act quickly and a precautionary requirement to prevent serious and irreversible harm, the importance of the *reversibility* of the precautions themselves increases (Nijsingh forthcoming). Although this formulation does not offer a clear cut-off point, reversibility could be interpreted as a threshold criterion: when, in a complex and urgent situation, the reversal of a precaution is impossible, likely to be very costly, or disruptive, this offers a weighty, principled reason against this precaution. As the available evidence accumulates, a situation may arise in which one may more confidently choose an option that is less easily reversed. However, our discussion suggests that the prospect of even retrospectively determining a single best course of action is not great, which means that it is important not to be overconfident in implementing precautions.

## Practical Implications

The focus on reversibility has several practical implications:

- Public policy should have sunset provisions and should clearly describe how the temporary character of the surveillance is to be guaranteed.
- Any responsible implementation of CT technology should give special weight to systemic risks, for example creating or exacerbating power asymmetries or the risk of creating or reinforcing monopolies.
- Reversibility provides an argument not to integrate CT technology into existing infrastructures: a keychain or card is prima facie preferable to an app that is installed on a smartphone. Specifically designated hardware may be easily discarded and plausibly has a relatively small impact on existing power distributions.
- Evidence should be acquired in local experiments, rather than nationally or even internationally rolling out possible solutions.
- The focus on reversibility also gives strong reasons not to presume effectiveness of CT apps in general. Different governments and private institutions have currently committed to digital CT, mostly without detailing the specific properties that would determine effectiveness, and without describing how effectiveness would be enhanced by other measures. This approach runs the risk of excluding considerations, which hinders a proper balancing of contextual factors.

Note that the fluidity of the current situation suggests that none of these implications is etched in stone and that they could themselves be subject to revision. Any plausible approach to the complex and urgent problems that we now face will, however, need to take reversibility into account.
